# Debridement of contaminated implants using air-polishing coupled with pH-responsive maximin H5-embedded metal-organic frameworks

**DOI:** 10.3389/fbioe.2023.1124107

**Published:** 2023-01-26

**Authors:** Yu Zhu, Qiang Zhi, Chunan Zhang, Yingxin Gu, Shuli Liu, Shichong Qiao, Hongchang Lai

**Affiliations:** ^1^ Department of Implant Dentistry, Shanghai Ninth People’s Hospital, College of Stomatology, Shanghai Jiao Tong University School of Medicine, Shanghai, China; ^2^ National Clinical Research Center for Oral Diseases, Shanghai, China; ^3^ Shanghai Key Laboratory of Stomatology and Shanghai Research Institute of Stomatology, Shanghai, China; ^4^ Department of Oral and Maxillofacial-Head and Neck Oncology, Shanghai Ninth People’s Hospital, College of Stomatology, Shanghai Jiao Tong University School of Medicine, Shanghai, China

**Keywords:** peri-implantitis, decontamination, air-polishing, antimicrobial agents, metal-organic frameworks

## Abstract

The primary goal of peri-implantitis treatments remains the decontamination of implant surfaces exposed to polymicrobial biofilms and renders biocompatibility. In this study, we reported a synergistic strategy for the debridement and re-osteogenesis of contaminated titanium by using erythritol air abrasion (AA) coupled with an as-synthesized pH-responsive antimicrobial agent. Here, the anionic antibacterial peptide Maximin H5 C-terminally deaminated isoform (MH5C) was introduced into the Zeolitic Imidazolate Frameworks (ZIF-8) *via* a one-pot synthesis process. The formed MH5C@ZIF-8 nanoparticles (NPs) not only possessed suitable stability, but also guarantee the slow-release effect of MH5C. Antibacterial experiments revealed that MH5C@ZIF-8 NPs exhibited excellent antimicrobial abilities toward pathogenic bacteria of peri-implantitis, confirming ZIF-8 NPs as efficient nanoplatforms for delivering antibacterial peptide. To evaluate the comprehensive debridement efficiency, single-species as well as mixed-species biofilms were successively established on commercially used titanium surfaces and decontaminated with different methods: removed only by erythritol air abrasion, treated merely with MH5C@ZIF-8 NPs, or received both managements. The results demonstrated that only erythritol air abrasion accompanied with MH5C@ZIF-8 NPs at high concentrations eliminated almost all retained bacteria and impeded biofilm rehabilitation, while neither erythritol air abrasion nor MH5C@ZIF-8 NPs alone could achieve this. Subsequently, we evaluated the re-osteogenesis on previously contaminated surfaces which were treated with different debridement methods afterwards. We found that cell growth and osteogenic differentiation of bone marrow–derived mesenchymal stem cells (BMSCs) in the group received both treatments (AA + MH5C@ZIF-8) were higher than those in other groups. Our work emphasized the great potential of the synergistic therapy as a credible alternative for removing microorganisms and rendering re-osseointegration on contaminated implant surfaces, boding well for the comprehensive applications in peri-implantitis treatments.

## 1 Introduction

Peri-implantitis has been defined as inflammatory reactions occurring in tissues around dental implants characterized by suppuration, bleeding, and bone loss, which ultimately lead to implant failures (Schwarz et al., 2018). The prevalence of peri-implantitis varied between 17% and 22% of patients and between 9.25% and 12.8% of the implant sites (Munoz et al., 2018). The main cause associated with peri-implantitis is the polymicrobial biofilms development owing to the settlement and expansion of pathogenic bacteria, including *F. nucleatum*, *P. gingivalis*, and *S. mutans* (Munoz et al., 2018). Therefore, the basic cause-related intervention for peri-implantitis remains the decontamination of implant surfaces exposed to bacterial biofilms and renders biocompatibility, with re-osseointegration as the ultimate objective. The screw-shaped designing and the microstructure surface of titanium implants may hinder the access of debridement instruments and limit the cleansing efficacy (Louropoulou et al., 2015). To date, no recognized gold standards for surface decontamination during peri-implantitis treatments have been suggested.

Mechanical decontamination involves scaling and polishing of implant surfaces. Treatment modalities such as polishing brushes and rubber cups fail to remove plaques from the depth of threads due to limited flexibilities (Louropoulou et al., 2015). Debridement managements such as curettes and ultrasonic scalers cause detrimental surface alterations and deposit residual fragments (Park et al., 2015). Promising results for air abrasion (AA) were concluded in a review and the authors demonstrated that “the cleaning efficiency of air abrasion on titanium strips, discs or implants is outstanding” (Park et al., 2015). The suspension of the air-powder system, made up of air abrasive powders and pressurized water-air mixture, is sprayed from the subgingival nozzles to implant surfaces, which would remove the plaque due to friction (Matsubara et al., 2020). The powder particles could be reflected in an angulation vertically to their angle of incidence, indicating that powders would reach these highly recessed areas (Keim et al., 2019). Compared with the classical glycine powders (25 μm) and sodium bicarbonate powders (40–65 μm), the erythritol powders (14 μm) were gentler to implant surface with no damage to the surface integrity (Matsubara et al., 2020). Moreover, the erythritol-based powders have higher dissolution potentials and more rapid degradation by the organisms (Drago et al., 2014). Nevertheless, merely mechanical decontamination with air-polishing remains a difficult task and depends mainly on the operator’s experiences.

Chemotherapeutic agents act as an adjunct to mechanical debridement, which weaken biofilms, facilitate its removal, and kill remaining bacteria. However, organic chemical agents including chlorhexidine (CHX) and hydrogen peroxide (H_2_O_2_) suffer from high toxicity and rapid release rate (Kotsakis et al., 2016). Local antibiotics including minocycline and tetracycline paste fail to decrease microorganisms compatible with peri-implant health and cause drug resistance (Ntrouka et al., 2011). Antimicrobial peptides (AMPs) which are ancient and effective antimicrobials of innate immune systems maybe an intelligent strategy (Dennison et al., 2018). The majority of AMPs are cationic, but a growing number of anionic AMPs (AAMPs) have attracted attentions (Dennison et al., 2018). The Maximin H5 (MH5) existing abundantly in the brain and the skin of the Chinese frog Bombina maxima is an AAMP with attractive characteristics (Dennison et al., 2016). MH5 owns membranolytic abilities, which mainly involves the bilayer insertion of the hydrophobic N-terminal region (Dennison et al., 2016). MH5 uses lipid interactions of the α-helical structure and exhibits antibacterial activities by the membranolytic method compared with the specific “Carpet” mechanisms (Dennison et al., 2015). There is strong evidence to suggest that MH5 kills gram-positive bacteria through the pH dependent membranolytic mode (Dennison et al., 2015). Nevertheless, MH5 exhibited limited activities against gram-negative bacteria (Dennison et al., 2015). Researches on AAMPs have demonstrated that C-terminal deamidation would strengthen the antibacterial effectiveness without enhancing the lyric capacity, hence promoting the therapeutic ability of the antimicrobial peptide (Ortiz-Gomez et al., 2020). Dennison et al. have described the effect of C-terminal deamidation on the antibacterial abilities of the peptide, and found that MH5 with the structure ILGPVLGLVSDTLDDVLGIL-COOH (MH5C) gained antibacterial abilities against those gram-negative bacteria (Dennison et al., 2015). These increased antimicrobial activities might be associated with hydrogen-bonding interactions occurring between the C-terminal amide structure of the peptide MH5C and the membrane surface of gram-negative bacteria (Dennison et al., 2015). Still, it is necessary to draw up a green and powerful nanoplatform to deliver the specific antibacterial peptide MH5C.

Metal−organic frameworks (MOFs) have attracted attentions in drug delivery due to porous structures, simple preparations, and multifunctional features (Karami et al., 2021; Li et al., 2022b). Among MOFs, zeotlitic imidazolate framework-8 (ZIF-8) constructed by zinc ions and 2-methylimidazole, serves as container of antibacterial metal ions and possesses the tailorable pore size for loading antimicrobial agents (Karami et al., 2021; Xia et al., 2022). It was found that ZIF-8 exhibited >99.9999% inactivation efficiency against gram-negative bacteria in saline under 2 h of simulated solar irradiation (Li et al., 2019). Another study demonstrated that ZIF-8 outperformed the extensively used antibacterial ZnO (Ahmed et al., 2019). The photoelectrons trapped within the zinc ions of ZIF-8 are responsible for ROS production inducing cell deformation and cytoplasm leakage of bacteria (Ahmed et al., 2019; Li et al., 2022a; Yu et al., 2022). It is noteworthy that the ZIF-8 crystal exhibits excellent capabilities for drug delivery because the ZIF-8 is stable in neutral or alkaline aqueous medium but break down rapidly in acidic medium (Liu et al., 2020a). The accumulating acetic acid and lactic acid in the inflammatory site cause low pH value, which was beneficial for specific release of ZIF-8 (Liu et al., 2020a). Previously the ZIF-8 was made in the dimethylformamide solution *via* the solvothermal process, but the dimethylformamide may be deposited within the pore space (Zou et al., 2020). Kida et al. Have mentioned the synthesis of the ZIF-8 crystal without by-products in pure water under room temperature *via* the one-pot method (Zou et al., 2020). The green synthesis method makes the ZIF-8 nanoparticles (NPs) appealing for the encapsulation of the fragile biomacromolecules including peptides, proteins, and enzymes. The peptide addition can modulate the shape and size of ZIF-8 crystals, which mainly depended on the molecular concentrations, biomolecule charges, and amino acid sequences. Some studies have affirmed that only negatively charged biomolecules with low isoelectric point (PI) would form biomolecules@ZIF-8, while biomolecules with neutral or positive charge would induce the development of a totally new phase *dia-Zn*(HmIm)_2_ (Carraro et al., 2020; Xuan et al., 2020). Thus the anionic antimicrobial peptides MH5C with negative charge (PI = 6.5) would make MH5C@ZIFs crystals frequently, boding well for its applications in chemical debridement.

In this work, we reported a synergistic strategy for the debridement and re-osteogenesis of contaminated titanium by using erythritol AA coupled with an as-synthesized pH-responsive antimicrobial agent. Our work not only highlights the great potential of applying ZIF-8 NPs as a green and robust nanoplatform to deliver antibacterial peptide in chemical decontamination, but also bring a new insight into the synergistic therapy for better managements of the contaminated dental implant surfaces during peri-implantitis treatments.

## 2 Materials and methods

### 2.1 Preparation of MH5C@ZIF-8

The MH5C-terminally deaminated isoform (MH5C) (ILGPVLGLVSDTLDDVLGIL-COOH, 95% purity) was synthesized by Top-peptide Co. (Shanghai, China). MH5C@ZIF-8 NPs and ZIF-8 NPs were conducted by the one-pot method at room temperature in water as previous studies (Li et al., 2018). As for the ZIF-8 NPs, dissolve zinc nitrate hexahydrate and 2-methylimidazole in deionized water, respectively. Then quickly add zinc nitrate solution into imidazole solution under the condition of stirring at room temperature. After complete crystallization (up to 12 h), a centrifuge (14,000 rpm, 10 min) was used to separate the solid and liquid of the solution. As for the MH5@ZIF-8 NPs, the aqueous solutions of MH5C and 2-methylimidazole was blended with the zinc nitrate aqueous solution through vigorous stirring. After completing crystallization (up to 12 h), the synthesized MH5C@ZIF-8 NPs were washed by the deionized water and then purified *via* centrifugation (14,000 rpm, 10 min). The purified MH5C@ZIF-8 and ZIF-8 NPs were completely freeze dried and kept at −20°C for the further experiments. Based on the equation that drug loading content (DLC, %) = (weight of loaded drug)/(weight of drug loaded nanoparticles) × 100% while drug loading efficiency (DLE, %) = (weight of loaded drug)/(weight of drug in feed) × 100%, the DLC and DLE of MH5C@ZIF-8 NPs was 9.8% and 33.2%, respectively.

### 2.2 Characterization of MH5C@ZIF-8

The scanning electron microscopy (SEM, Hitachi S-4800, Japan) and transmission electron microscopy (TEM, Tecnai G2, United States) were utilized to characterize the morphology. The dynamic light scattering (DLS, Nicomp 380, United States) was utilized to measure the size distributions. The powder X-ray diffraction (XRD, D8 Advance A25, United States) was utilized to characterize the crystalline structure. The fourier transform infrared (FTIR, Nicoletteis50, United States) was utilized to explore the chemical structure. The thermogravimetric analyzer (TGA, Mettler Toledo, Switzerland) was utilized to complete the thermogravimetric study from 20°C to 820°C under air atmosphere. The Malvern Zeta Sizer-Nano ZS90 instrument was utilized to measure the zeta potential. As for the release of MH5C, the BCA reagents (Beyotime, China) was utilized to determine the peptide concentration and the release behavior of MH5C@ZIF-8 was accomplished in both PBS buffer at pH 7.4 and MES buffer at pH 5.5.

### 2.3 Microbial and cellular culture

The bacterial strains *F. nucleatum* (ATCC10953), *P. gingivalis* (ATCC 33277), and *S. mutans* (UA 159) were acquired from the Shanghai Key Laboratory of Stomatology, Ninth People’s Hospital, affiliated with Shanghai Jiao Tong University, School of Medicine. Bacteria were seeded on the brain heart infusion (BHI) agar in the presence or absence of sterile sheep blood. After gram staining routinely, the single colony was chosen and cultured in the BHI broth. All species were culture in an anaerobic incubator. The bacteria of the log phase with vigorous activities were applied for further use.

The single-species and mixed-species biofilms was developed directly on the 24-well culture plate or on the surface of sandblasted, large-grit and acid-etched (SLA) titanium discs (15 mm in diameter, 1 mm in thickness). The bacterial suspensions containing 10^4^ colony-forming units (CFU)/mL for *F. nucleatum*, 10^6^ CFU/mL for *P. gingivalis*, and 10^5^ CFU/mL for *S. mutans* were utilized separately or all together to provide the single-species and mixed-species biofilms. The above plates or titanium disks were cultured for two consecutive days to develop biofilms as previous studies (Qin et al., 2020).

The 4-week-old SD rats were utilized to acquire rat bone marrow–derived mesenchymal stem cells (rBMSCs), which was approved by the Animal Care and Experiment Committee of Shanghai Ninth People’s Hospital affiliated to Shanghai Jiao Tong University (Protocol Number: SH9H-2020-A612-1). After separating both ends of the femora, the marrow was flushed out. The isolated cells were cultured with DMEM with 10% FBS in an incubator. Then 72 h later, non-adherent BMSCs were removed by utilizing the PBS buffer. Once reaching 80%–90% confluence, cells were sub-cultured to 2-3 dishes. The 2–3 passages of BMSCs were adopted for the following experiments.

### 2.4 Antibacterial activity of MH5C@ZIF-8

The growth curve indicating the bacterial growth in different systems was drawn up *via* the record of the OD value for different bacteria suspensions with time. Here, the bacterial concentration of *F. nucleatum*, *P. gingivalis* and *S. mutans* was initially set at approximately 10^8^ CFU/mL, corresponding to the OD value at 0. 135–0150 for these three species. To study the slow-release sterilization ability of MH5C@ZIF-8 NPs, pure MH5C and MH5C@ZIF-8 with the same peptide concentration were added into the bacterial solutions. As for pure MH5C, the MH5C concentrations were 31.25, 62.5, 125, and 250 μg/mL. As for MH5C@ZIF-8 NPs, the final equivalent concentration of peptide MH5C varied from 31.25 to 250 μg/mL. The bacterial suspension without antibacterial systems was applied as the control. The OD values at 600 nm within both antibacterial materials were recorded at regular time interval, until the growth curve of the specific bacterial species have reached the plateau.

The 3-(4,5-dimethylthiazol-2-yl)-2,5-diphenyl tetrazolium bromide (MTT) assay was used to evaluate the relative bacterial viability. In brief, the mixed-species biofilms were developed directly on the conventional 24-well culture plate instead of titanium discs. The bacterial suspensions were discarded and the retained biofilms were treated with pure MH5C or MH5C@ZIF-8 for 12 h and 24 h. Then the previous liquid was discarded and the MTT (Beyotime, China) solution was added. After 2 h in the dark, the unreacted MTT was removed and dimethyl sulfoxide (DMSO) was utilized for dissolving the formazan crystal. Afterwards, the above solution was transferred to the 96-well plate and OD at 490 nm was recorded through the BioTek instrument.

The biomasses of the bacterial biofilms were determined *via* the crystal violet (CV) staining assay. In brief, the mixed-species biofilms were developed directly on the culture plate and the biofilms were treated with pure MH5C or MH5C@ZIF-8 for 12 h and 24 h. Then the previous liquid was discarded and 100 μL of 0.01% (v/v) CV (Sigma, United States) solution was added to each well for 15 min. After washed with PBS, each well was added with ethanol. Afterwards, the above solution was transferred to the 96-well plate and OD at 595 nm was recorded through the BioTek instrument.

### 2.5 Cleaning capacity of mechanical-chemical synergistic treatments

To evaluate the decontamination effects of the synergistic therapy, we designed four experimental groups: group AA, biofilms removed with erythritol air abrasion (AA); group MH5C@ZIF-8, biofilms dealt with high concentrations of MH5C@ZIF-8 (125 μg/mL) for 24 h; group AA + MH5C@ZIF-8, biofilms treated with MH5C@ZIF-8 (125 μg/mL) for 24 h followed by erythritol AA; and the control group, untreated biofilms. Specifically, the single-species and mixed-species biofilms were developed directly on the SLA titanium discs. After washed with PBS to eliminate the unattached bacteria, the contaminated titanium discs were transferred to the 24-well culture plate. Then the fresh BHI medium with (group MH5C@ZIF-8 and AA + MH5C@ZIF-8) or without (groups Control and AA) the MH5C@ZIF-8 solution (125 μg/mL) was introduced into each well. After 24 h, for groups AA and AA + MH5C@ZIF-8, the biofilms on titanium discs in group AA and AA + MH5C@ZIF-8 were cleaned by the AA device (EL-308/C, EMS Nyon, Switzerland) with erythritol powders (particle size ≈14 mm) under the static pressure (7 bar) for totally 1 min, perpendicular to titanium discs, at a distance of 10 mm. Here, all discs have received treatments by being continuously rotated opposing the subgingival nozzles from center to periphery in four different circular motions. After air abrasions, all discs were dried by the compressed air for nearly 10 s for removing the retained powders. All air abrasions were finished by the same experienced operator. Afterwards, the titanium plates in each group were washed by PBS, transferred to the new 24-well plate, and received the subsequent antimicrobial assessments.

The single-species biofilm integrity after decontamination methods was detected by SEM examinations (JSM-7600F, Japan). In detail, samples were rinsed by PBS and fixed by using glutaraldehyde at room temperature for 2 h. Then samples were dehydrated by the ethanol concentration gradient, ending with the 100% ethanol for 30 min. Before visualization, each sample was dried and sputter coated with gold. SEM images were taken at different locations on each sample.

The single-species biofilm bioactivity after decontamination methods was detected by the live/dead fluorescence staining. The staining solution included 2.5 mM SYTO9 and 2.5 mM propidium iodide. The intact bacteria were stained with SYTO9 emitting the green fluorescence, and the membrane-compromised bacteria were stained with propidium iodide emitting the red fluorescence. After staining in the dark for 15 min, fluorescence images on each sample were collected with the CLSM (Olympus FV1000, Japan).

The bacterial viability and biomasses of the mixed-species biofilms after decontamination methods were determined by the MTT assay and the CV staining assay, respectively. Here, the mixed-species biofilms were developed directly on the SLA titanium discs. After different debridement, the remaining biofilms in each group were assessed by the MTT assay and the CV staining assay according to the methods mentioned above.

### 2.6 Biofilm removal assessment using implant models

Six cylindric tissue-level titanium implants with SLA surfaces (3.3 mm in diameter, 12 mm in height) were allocated to two groups. The mixed-species biofilms were produced on implant surfaces by immersing implants into mixed bacterial suspensions. The group AA + MH5C@ZIF-8 received the MH5C@ZIF-8 (125 μg/mL) pre-treatment for 24 h, whereas the group AA was merely immersed into the fresh BHI medium. Subsequently, each implant was evenly coated by an intact layer of bacterial indicator for recognition of the aggregated plaque following the manufacture’s recommendations. Afterwards, all implants received the identical erythritol air abrasion treatment by the same experienced clinician *via* the AA device with subgingival nozzles (EMS, Switzerland) (Sahrmann et al., 2015; Matsubara et al., 2020). The distance was set to 10 mm, the static pressure was set at 7 bar, and the processing time was set to totally 1 min. After air abrasions, all discs were dried by the compressed air for nearly 10 s for removing the retained powders.

As for biofilm removal assessment, the standard digital photographs of dental implants were taken with the digital SLR camera (Canon digital SLR). Here, the shutter speed was set at 1/4,000 and the aperture was set at F32. These photos have been compounded so as to build the panoramic images of dental implants. With the aid of the color analysis software (Photoshop CC, United States), the pixel percentages of retained plaque indicators were assessed and the corresponding percentage of biofilm removal was then calculated. As for the implant surfaces analysis, the area roughness (Sa) of implant surfaces was measured by the optical profilometry analysis through the TrueSurface Microscopy (WITec Alpha 300, Germany). Specifically, the Sa value was obtained *via* the evaluations of change in height on implant surfaces across numerous individual points.

### 2.7 Re-osseointegration potential after debridement

To evaluate the re-osseointegration potentials of the contaminated titanium surface after debridement, five experimental groups were designed for further cellular experiments: group AA, biofilms removed with erythritol air abrasion (AA); group MH5C@ZIF-8, biofilms dealt with high concentrations of MH5C@ZIF-8 (125 μg/mL) for 24 h; group AA + MH5C@ZIF-8, biofilms treated with MH5C@ZIF-8 (125 μg/mL) for 24 h followed by erythritol AA; the control group, untreated biofilms; and the clean group, totally intact titanium discs without biofilms and never contaminated by bacteria suspensions. Before subsequent experiments, all titanium discs were sterilized by the ethylene oxide sterilizer. Hence merely dead biofilms or bacteria were retained on titanium surfaces.

As for cell adhesions, cell suspensions with the cell density at 1 × 10^4^ cell/mL was transferred to each sample. After cultured for 24 h, rBMSCs were fixed in paraformaldehyde for 10 min and then permeabilized by 0.5% Triton X-100 for nearly 5 min. Afterwards, each sample was firstly stained by fluorescein isothiocyanate-phalloidin (FITC, Sigma-Aldrich, United States) for 30 min and further stained by the 4′,6-diamidino-2-phenylindole dihydrochloride (DAPI, Sigma-Aldrich, United States) for 5 min in the dark. The fluorescence images were obtained *via* the inverted fluorescence microscope (IX70, Olympus Corporation, Japan).

As for cell proliferation, cell suspensions with the cell density at 1 × 10^4^ cell/mL was transferred to each sample initially. Then the rBMSCs viability was evaluated by the cell counting kit (CCK)-8 assay (AbD Serotec, UK) at 1, 3, and 7 days. Specifically, CCK-8 solution was transferred to different samples and cultured for totally 2 h, and the absorbance at 450 nm was recorded by the BioTek instrument.

As for cell osteogenic differentiations, cell suspensions with the cell density at 4 × 10^4^ cell/mL was transferred to each sample initially. After 24 h, the previous culture medium was changed to the differentiation medium containing DMEM supplemented with dexamethasone, sodium β-glycerophosphate and ascorbic acid-2-phosphate (Thermo Fisher Scientific, United States). The staining of alkaline phosphatase (ALP) was carried out at 4, 7, and 14 days. In brief, samples were fixed in paraformaldehyde and stained by using the BCIP/NBT ALP Color Development Kit (Beyotime, China). Total RNA of rBMSCs at 7 and 14 days was extracted by the TRIzol reagent (Thermo Fisher Scientific, United States) and then reverse transcribed into cDNA by the Primescript RT Reagent Kit (TakaraBio Inc., Japan). Then the expressions of the osteogenesis-related genes were determined by the reverse transcription-polymerase chain reaction (RT-PCR) (LightCycler 480, Switzerland) *via* the QuantiTest SYBR Green Kit (Takara, Japan). Here, β-actin was used as an internal control. The relative ratios were analyzed by the relative expression analysis (2^−ΔΔCT^). The primer sequences were displayed in Table 1.

**TABLE 1:** Primer pairs used in real-time PCR analysis.

Gene	Primers (F = forward, R = reverse)
ALP	F: TCCGTGGGTCGGATTCCT
	R: GCCGGCCCAAGAGAGAA
OPN	F: TCC​AAG​GAG​TAT​AAG​CAG​CGG​GCC​A
	R: CTC​TTA​GGG​TCT​AGG​ACT​AGC​TTC​T
RUNX2	F: ACC​AGC​AGC​ACT​CCA​TAT​CTC​TAC
	R: CTT​CCA​TCA​GCG​TCA​ACA​CCA​TC
OCN	F: ATT​GTG​ACG​AGC​TAG​CGG​AC
	R: GCA​ACA​CAT​GCC​CTA​AAC​GG
β-actin	F: CAC​CCG​CGA​GTA​CAA​CCT​TC
	R: CCC​ATA​CCC​ACC​ATC​ACA​CC

### 2.8 Statistical analysis

Each experiment was performed in triplicates, and the data was expressed as means ± standard deviations. The one-way analysis of variation coupled with the Student–Newman–Keuls *post-hoc* tests was utilized to assess the level of significance. The significance level was set at *p < 0.05.

## 3 Results and discussion

### 3.1 Synthesis and characterization of nanoparticles

**FIGURE 1:**
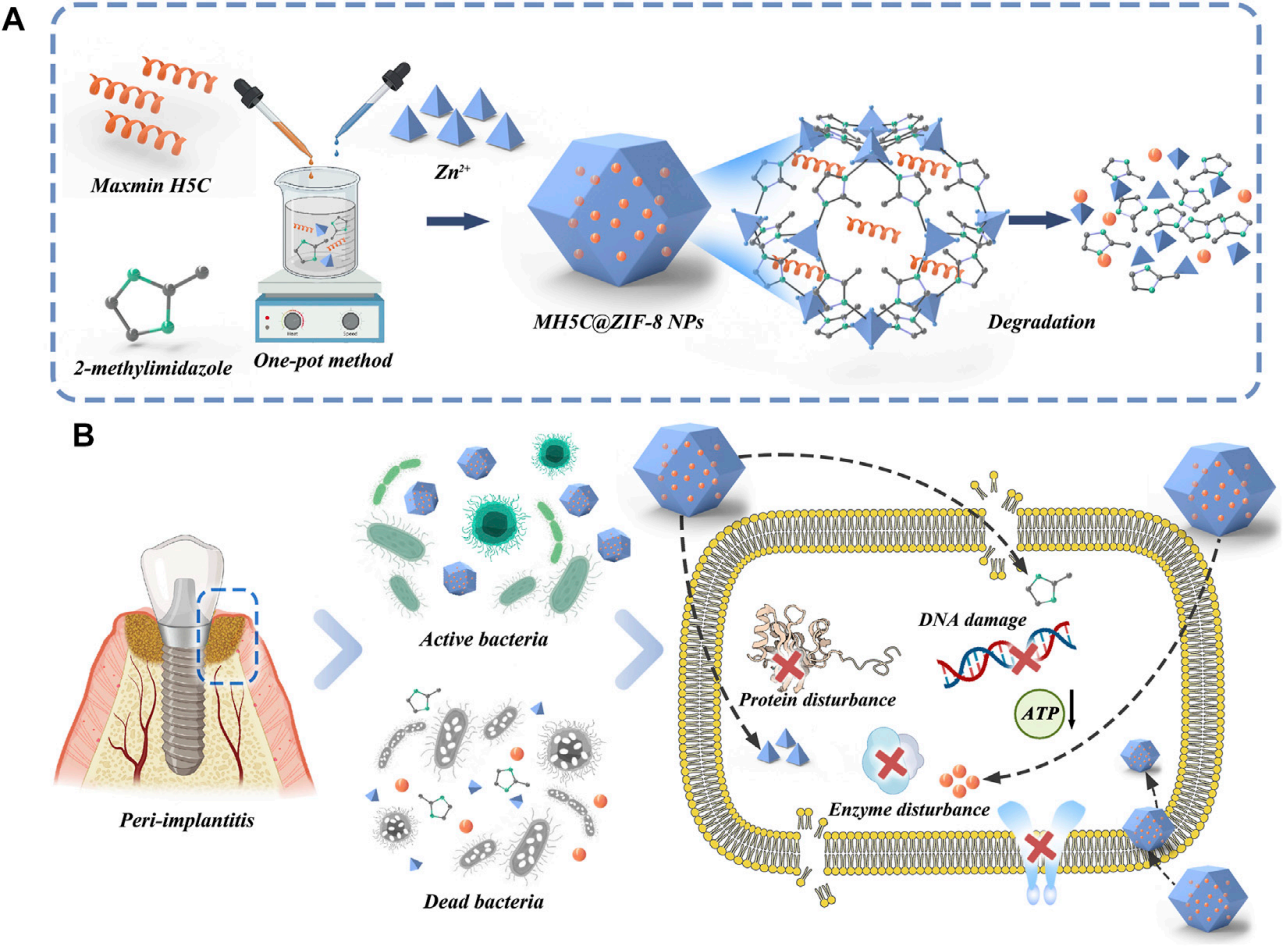
**(A)** Schematic illustration of the fabrication of MH5C@ZIF-8 NPs and degradation route after entering bacteria. **(B)** Potential antimicrobial mechanisms involved in this chemical decontamination strategy.

The native structure of antimicrobial peptide MH5 was ILGPVLGLVSDTLDDVLGIL-NH2 (MH5N) (Ortiz-Gomez et al., 2020). The peptide MH5C (ILGPVLGLVSDTLDDVLGIL-COOH), the C-terminally deamidated isoform of MH5, was a hydrophobic anionic molecule with 20 amino acids (Ortiz-Gomez et al., 2020). Dennison et al. (2015) have demonstrated the significant effects of C-terminal deamidation on the antibacterial activities of peptide MH5 against gram-negative bacteria. During the typical biomimetic mineralization, biomolecules (including antimicrobial peptide) were trapped into ZIF-8 NPs, formed by the self-assembly of organic linkers and metal ions through the facile one-pot water phase method (Abdelhamid, 2021). The synthetic procedure of ZIF-8 and MH5C@ZIF-8 NPs were schematically illustrated in Figure 1A. Specifically, an aqueous solution containing the target antimicrobial peptide MH5C together with 2-methylimidazole was blended with the aqueous solution of the zinc nitrate. It is worth mentioning that the synthetic procedure here was green, simple, and rapid. In the synthesis processes, the synergistic interactions between biomolecules and precursors were extremely crucial to the nucleation and growth process (Xuan et al., 2020; Zou et al., 2020; Han et al., 2022). Previous studies have also found that only biomolecules with negative charge or with low isoelectric point (PI) would form biomolecules@ZIF-8 (Fan et al., 2018; Maddigan et al., 2018; He et al., 2019). In this study, the MH5C with the PI of 6.5, which exhibited negative charge at the neutral pH, has been utilized for the formation of biomimetically mineralized ZIF-8. During mineralization, the initial indication for nanoparticles formations was the transformation of transparent solutions containing reactants into turbid solutions following vigorous stirrings (Xuan et al., 2020; Zou et al., 2020). As expected, the biomineralization process of MH5C under the specific reaction conditions was very quick (within several minutes). Based on these, it was estimated that the negatively charged peptide MH5C posed specific affinities to the specific ZIF-8 precursor owing to electrostatic attractions, which would finally induce the rapid form of MH5C@ZIF-8 NPs.

**FIGURE 2:**
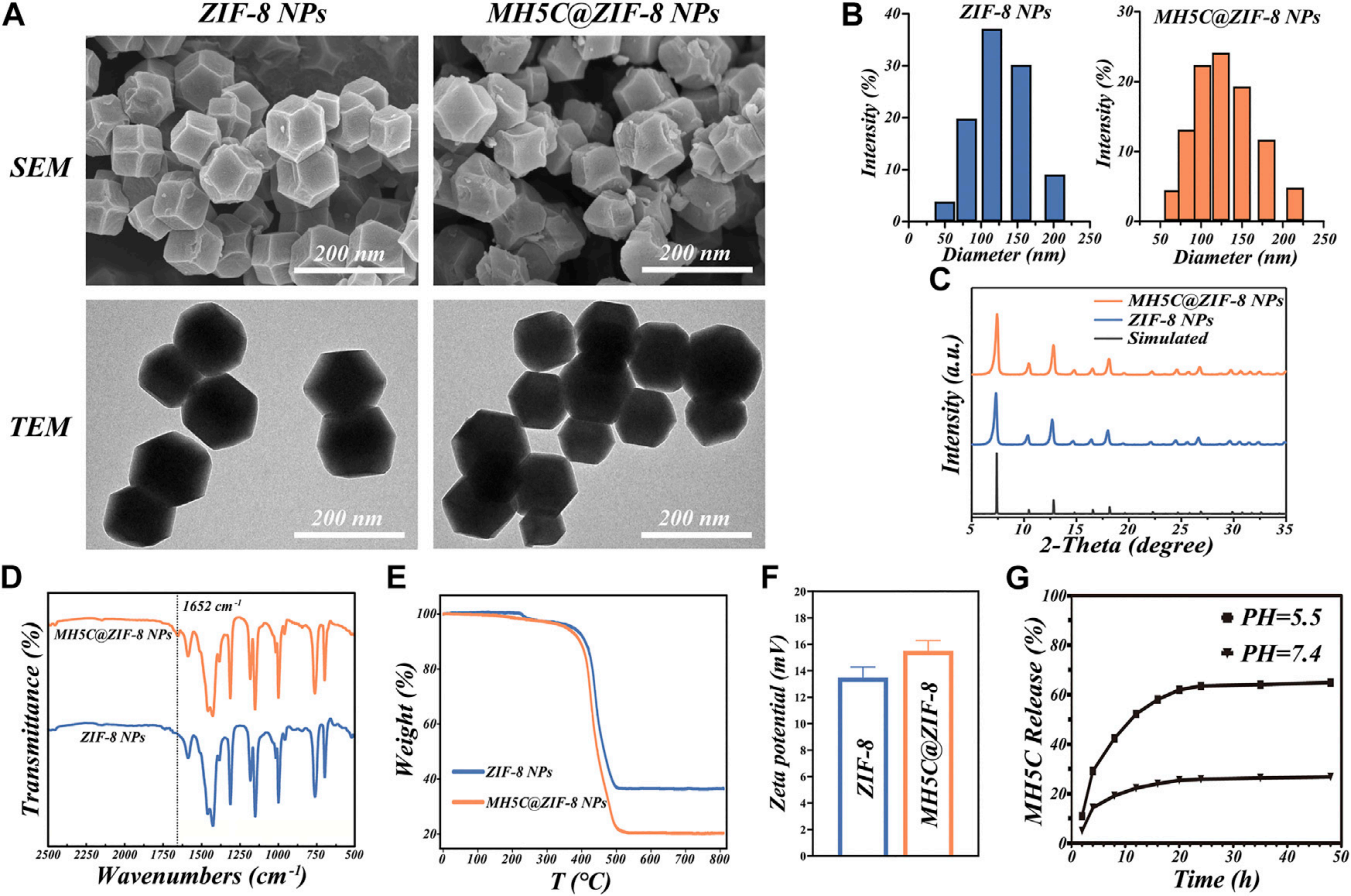
Basic properties of MH5C@ZIF-8 NPs. **(A)** SEM and TEM images of MH5C@ZIF-8 NPs and ZIF-8 NPs. **(B)** Particle size distribution of MH5C@ZIF-8 NPs and ZIF-8 NPs dispersed in water. **(C)** XRD patterns of MH5C@ZIF-8 NPs, ZIF-8 NPs and simulated ZIF-8. **(D)** FTIR spectra of MH5C@ZIF-8 NPs and ZIF-8 NPs. **(E)**TGA curves of MH5C@ZIF-8 NPs and ZIF-8 NPs. **(F)** Zeta potential of ZIF-8 NPs and MLT@ZIF-8 NPs. **(G)** MH5C release profiles from MH5C@ZIF-8 in PBS solution (pH = 5.5 and 7.4).

In order to gain a deeper understanding of the microstructure of MH5C@ZIF-8 NPs and its ZIF-8 NPs control, samples were observed by SEM, which revealed that both monodisperse particles possessed a regular rhomboidal dodecahedron shape accompanied with a relatively smooth surface (Figure 2A). The TEM images depicted the macroscopic state of nanoparticles in water. The ZIF-8 NPs exhibited the rhombic dodecahedron structures accompanied by the sharp edge as well as the uniform particle sizes, whereas encapsulation of MH5C into ZIF-8 seemed to slightly smooth edge sharpness of these dodecahedrons and increase the diameters of these nanoparticles fractionally (Figure 2A). It is presumed that the loading of MH5C did not affect the shape of ZIF-8 NPs, indicating that MH5C were confined in the interior micropore or entrapped on the surface of ZIF-8 frameworks (Wen et al., 2021). Based on the DLS analysis, the hydrodynamic diameter of MH5C@ZIF-8 NPs displayed the relative narrow size distributions (124.6 ± 4.7 nm) that was slightly larger than that of the ZIF-8 control (107.8 ± 5.1 nm), indicating that the growth process of ZIF-8 was scarcely influenced by the incorporation of MH5C (Figure 2B). Overall, as compared with ZIF-8 NPs, no significant differences were observed in the size and morphology of MH5C@ZIF-8 NPs, suggesting that ZIF-8 would be employed as the promising nanocarrier in the delivery of the antimicrobial peptide MH5C.

The crystalline structure of MH5C@ZIF-8 and pure ZIF-8 NPs was assessed by the XRD measurement. The XRD pattern of the ZIF-8 control here was identical to the simulated one, verifying the smooth formation of the crystal structure (Figure 2C). Specifically, the diffraction peaks at 7.3°, 10.7°, 12.7°, 14.6°, 16.4°, 18.0°, 22.1°, 24.5°, and 26.7° of ZIF-8 control were corresponded to the lattice planes of ZIF-8 [(011), (002), (112), (022), (013), (222)), (114), (233) and (134)] (Yang K. et al., 2022). After the loading of MH5C, it is worth noting that the XRD patterns of MH5@ZIF-8 NPs coincided with those of pure ZIF-8 NPs, suggesting that the crystalline structures of ZIF-8 were not influenced by the encapsulation processes of the MH5C, which was in consistency with the results of electron microscopy (Figure 2C). The chemical structure and functional group of MH5C@ZIF-8 and ZIF-8 NPs were explored by FTIR spectrum. For the ZIF-8 control, the characteristic peaks at 1,585 and 420 cm^−1^ were ascribed to the C=N bond and Zn-N stretching, while the peaks at 1,500–1,350, 1,350–900 and 900–650 cm^−1^ were attributed to the entire ring stretching, in-plane- and out-plane-bending of the ring, which belonged to the pure ZIF-8 (Figure 2D) (Xu et al., 2020; Wen et al., 2021; Yang K. et al., 2022). After the loading of the peptide MH5C, the new absorption bond at 1,652 cm^−1^ agreeing with carbonyls groups of MH5C was observed in the MH5C@ZIF-8 FTIR spectrum, verifying the existence of MH5C within the nanoparticles (Figure 2D) (Li et al., 2018). The TGA was utilized to evaluate the thermal characteristics and loading contents. From the TGA curve under air atmosphere, the minimal weight loss occurred initially at approximately 100°C–200°C, mainly originating from the removals of inorganic salts, water, and the small guest molecule (Figure 2E) (Hao et al., 2022). The subsequent weight loss stage appeared in the temperature of 400°C, and the weight loss became more notable with the increase of temperature, illustrating the gradual destroy of the ZIF-8 skeletons (Hao et al., 2022). The decomposition lasted until reaching approximately 500°C when ZIF-8 has been almost completely degraded, and the remaining materials was carbon and zinc oxide (Hao et al., 2022). The TGA curves of MH5C@ZIF-8 were similar to that of pure ZIF-8, but the weight loss extent of MH5C@ZIF-8 was more notable and the difference was calculated as approximately 13.1 wt%, which resulted from the decompositions of the peptide MH5C embedded within nanoparticles (Figure 2E). As shown in Figure 2F, the surface zeta potential value of pure ZIF-8 and MH5C@ZIF-8 NPs are +13.5 ± 0.77 mV and +15.5 ± 0.81 mV, respectively, verifying that the vast majority of the peptide MH5C might be embedded into the frameworks of ZIF-8 (Li et al., 2018). The slight increase in zeta potential might be due to the normally restricted part of electronegative MH5C absorbed onto the external surface of crystal structures (Li et al., 2018; Hao et al., 2022).

It is widely assumed that coordination between 2-methylimi-dazolate and zinc ions was acid-sensitive, ZIF-8 NPs were stable under neutral conditions but degrade rapidly in the acidic environment (Yu et al., 2021). To demonstrate the potentials of MH5C@ZIF-8 NPs as a pH-responsive delivering nano-systems of antimicrobial peptide, the release study of MH5C was completed in both PBS buffer at pH 7.4 and MES buffer at pH 5.5. The releases of MH5C from MH5C@ZIF-8 NPs were inefficient and the release rate accounted for about 25% in the PBS within 24 h, indicating the stability in the normal physiological environment and the specific property would inhibit the early leakage of peptide during circulations (Figure 2G) (Meng et al., 2022). In contrast, MH5C released rapidly from MH5C@ZIF-8 and the release rate accounted for as over 65% at PH 5.5 within 24 h, indicating that the peptide could be released more rapidly in the low pH conditions similar to inflammatory reactions environments in our bodies (Figure 2G) (Meng et al., 2022). The release of peptide from MH5C@ZIF-8 in a low pH environment was related to the decompositions of ZIF-8 skeletons through the breakages of the coordinate bonds between imidazolate and zinc ions. Hence, it was speculated that the MH5C@ZIF-8 particle was a quite promising pH-sensitive delivering nano-system of peptide for treatments of bacterial infection around dental implants due to the acidic environment.

### 3.2 Antibacterial activity of nanoparticles

**FIGURE 3:**
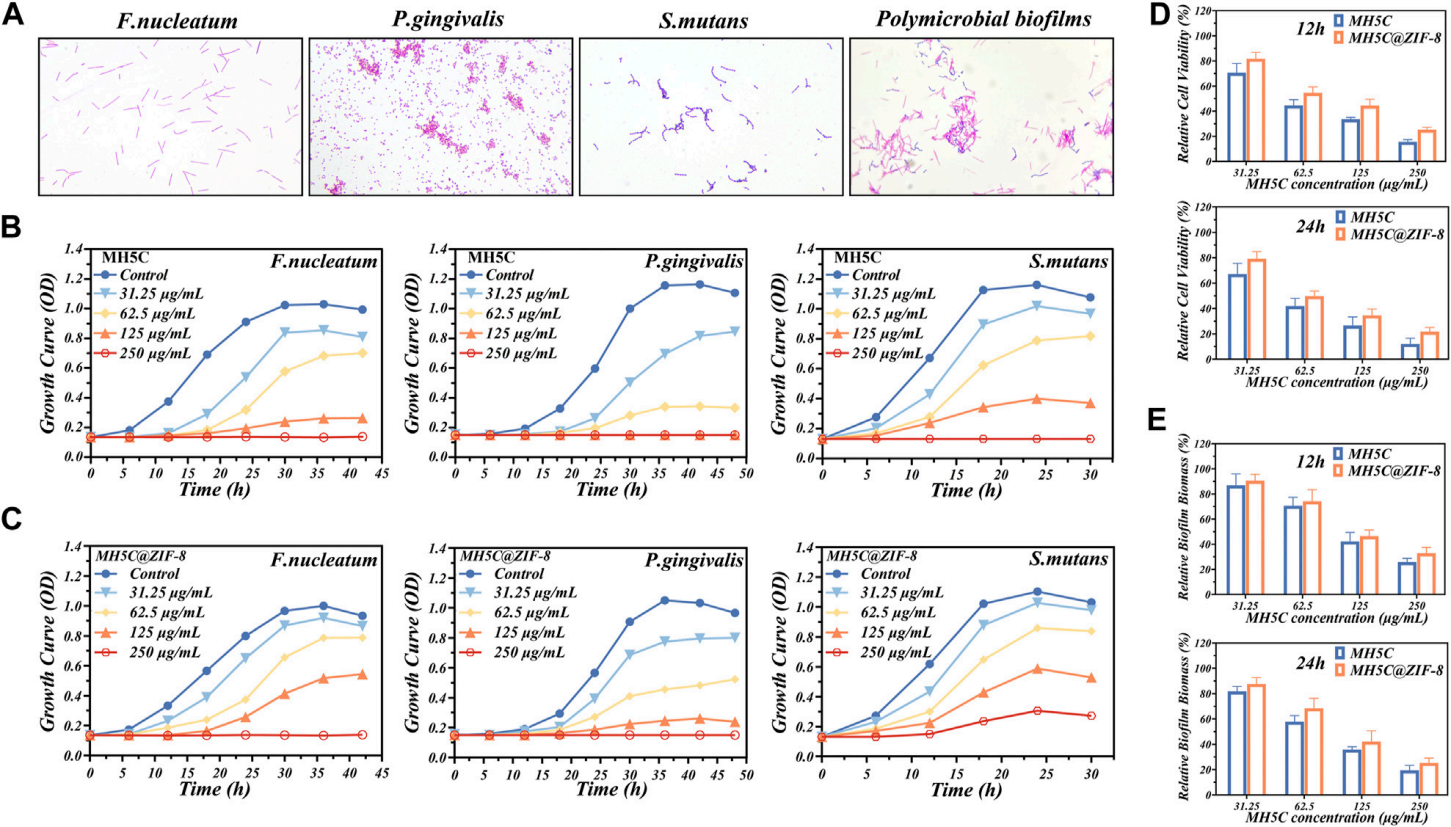
Antibacterial properties of MH5C@ZIF-8 NPs. **(A)** Gram staining of *F. nucleatum*, *P. gingivalis*, *S. mutans*, and polymicrobial biofilm. **(B)** Growth curves of the three species of bacteria in free MH5C with different concentrations. **(C)** Growth curves of the three species of bacteria in MH5C@ZIF-8 NPs with different concentrations. **(D)** Bacterial activity in the presence of free MH5C and MH5C@ZIF-8 NPs with different concentrations after 12 h and 24 h. **(E)** Biofilm biomass in the presence of free MH5C and MH5C@ZIF-8 NPs with different concentrations after 12 h and 24 h.

Oral microbes related to peri-implantitis were variable, and most of the time, dominated by the gram-negative anaerobic bacterium, involving *F. nucleatum* and *P. gingivalis* (Zhu et al., 2017). In addition, gram-positive *S. mutans* showed high affinity to the titanium surface (Zhu et al., 2017). Previous studies have reported that *P. gingivalis* bound with *S. mutans* through the incorporations of *F. nucleatum*, which generated the polymicrobial biofilm mimicking the peri-implantitis conditions (Guo et al., 2017; Qin et al., 2020). Figure 3A showed the gram staining of bacteria from the three single specie or the polymicrobial biofilm: the gram-positive *S. mutans* were the purple cocci in chains; the gram-negative *P. gingivalis* were the red cocci; and the gram-negative *F. nucleatum* were the red rod-shaped cells.

In most cases, bacteria would experience four stages, comprising lag phase, log phase, stationary phase, and death phase (Qin et al., 2020). According to the growth curve of the three bacterial strains, bacteria would reach the log phase after about 12 h for *F. nucleatum*, 24 h for *P. gingivalis*, and 6 h for *S. mutans* (Figure 3B). The three strains were harvested when they reached the log phase, because bacteria at this phase were in an optimal growth state corresponding to highly proliferative capabilities for the formation of mature biofilms (Lu et al., 2014; Cao et al., 2020). Here, the antibacterial abilities of the peptide MH5C were reflected by the time spent to enter the specific log phase. The growth curves of three bacterial strains cultured in a series of concentrations of MH5C solutions were shown in Figure 3B. As expected, the growth of *F. nucleatum* slowed down and the amount of *F. nucleatum* during the stationary phase were decreased obviously with the increase of the MH5C concentration. Especially, when the concentration of MH5C reached 125 μg/mL, the OD values of *F. nucleatum* remained unchanged across the testing periods from beginning to end, suggesting that almost all bacteria were killed during the lag stage. Moreover, after the stationary stage, the amount of *F. nucleatum* reduced gradually, which resulted from the facts that nutrient in MH5C-contained medium could not sustain the life of bacteria any longer. The overall growth trends of *P. gingivalis* as well as *S. mutans* were similar to that of *F. nucleatum*, and the time to enter the log phase was highly associated with the peptide concentration (Figure 3B). These phenomena indicated the concentration-dependent antibacterial activities of MH5C, which were consistent with previous studies (Dennison et al., 2015; Ortiz-Gomez et al., 2020).

To assess the slow-release sterilization properties of MH5C@ZIF-8 nano-systems, the MH5C@ZIF-8 NPs with specific concentrations of MH5C were added to the culture medium. The growth curves of three bacterial strains cultured in a series of concentrations of MH5C@ZIF-8 solutions were shown in Figure 3C. As compared to the blank culture medium, bacteria in solutions containing ZIF-8 NPs were decreased slightly, confirming the fact that pure ZIF-8 NPs inhibited the growth of *P. gingivalis*, *F. nucleatum*, and *S. mutans* (Figure 3C). Previous studies have shown that photoelectrons trapped at the center of zinc ions were responsible for the production of reactive oxygen species *via* ligand to metal charge transfer, finally inducing cell deformations, cell wall ruptures and cytoplasm leakages (Ahmed et al., 2019; Taheri and Tsuzuki, 2021). In comparison with pure MH5C, the antimicrobial activities of MH5C@ZIF-8 against three bacteria strains were more or less weakened (Figure 3C). These results might be ascribed to the slow-release rates of MH5C in the MH5C@ZIF-8 nano-systems and the maximum utilizations of antimicrobial capacities of MH5C@ZIF-8 NPs (Cao et al., 2020). In detail, quite a limited part of the MH5C was released to the solutions in the beginning, for the released antibacterial peptide in the first 24 h accounted for merely 25% of the total released antibacterial peptide. Nevertheless, the amounts of MH5C from MH5C@ZIF-8 were relatively enough to prevent the bacteria growth during the initial period (Figure 3C). Besides, the MH5C encapsulated in the ZIF-8 framework might have longer sterilization time than pure MH5C in the liquid phase, in other words, the MH5C@ZIF-8 might possess controllable antibacterial activity against the three bacteria stains (Cao et al., 2020). After comparing Figures 3B, C, the order of the three species easy to be killed by both pure MH5C and MH5C@ZIF-8 was *P. gingivalis*, *F. nucleatum*, and *S. mutans*. The increased antimicrobial activities against gram-negative pathogens might be owing to the direct hydrogen-bonding interaction occurring between the bacteria membranes of gram-negative bacteria and the C-terminal amide groups of the peptide MH5C (Dennison et al., 2015; Ortiz-Gomez et al., 2020).

After cultivation for 2 days, the polymicrobial biofilms were formed on the surface of culture plates. To evaluate the antimicrobial effects of pure MH5C and MH5C@ZIF-8 NPs on polymicrobial biofilms, the MTT assay and the CV staining assay were used to evaluate the biofilm biomass and cell the viability, respectively. After 12 h, the relative cell viability in group MH5C with MH5C concentrations of 31.25, 62.5, 125, and 250 μg/mL was 70.57% ± 7.45%, 44.57% ± 4.67%, 33.63% ± 1.43%, and 15.60% ± 1.68%, whereas that in group MH5C@ZIF-8 was 81.82% ± 5.02%, 54.60% ± 4.65%, 44.46% ± 5.04%, and 25.31% ± 1.90% (Figure 3D). After 24 h, the relative cell viability in group MH5C was 67.15% ± 8.45%, 41.98% ± 6.11%, 26.61% ± 6.75%, and 12.09% ± 4.43%, whereas that in group MH5C@ZIF-8 was 79.23% ± 5.68%, 49.76% ± 4.01%, 34.61% ± 5.03%, and 21.76% ± 3.37% (Figure 3D). After 12 h, the relative biofilm biomass in group MH5C was 86.87% ± 9.11%, 70.65% ± 6.68%, 42.24% ± 717%, and 25.83% ± 2.93%, whereas that in group MH5C@ZIF-8 was 90.56% ± 5.11%, 74.25% ± 9.21%, 46.45% ± 4.84%, and 32.87% ± 4.58% (Figure 3E). After 24 h, the relative biofilm biomass in group MH5C was 81.78% ± 3.97%, 57.86% ± 4.81%, 35.87% ± 2.19%, and 19.46% ± 3.83%, whereas that in group MH5C@ZIF-8 was 87.65% ± 4.96%, 68.47% ± 7.90%, 42.13% ± 8.49%, and 25.35% ± 3.75% (Figure 3E). These antibiofilm results demonstrated that the bacterial viabilities and the biofilm biomass decreased as the MH5C concentrations increased, confirming that the antibiofilm property of MH5C was concentration dependent. Moreover, antibiofilm results quantitatively demonstrated that the biofilm destruction efficiencies were slightly decreased as MH5C encapsulated into the ZIF-8 crystals, partly due to the gradual degradation of ZIF-8 and the sustained-release of MH5C (Yu et al., 2021). The slow-release sterilization abilities were mainly associated with degradation of MOFs, further determined by the chemical stability and the structural characteristic of MOFs (Cao et al., 2020). It is widely accepted that too strong stabilities made it tough to release antibacterial peptides, whereas too weak stabilities caused rapid release rates and underutilizations of antibacterial peptides (Yang M. et al., 2022). Our results showed that the structural stability of as-synthesized MH5C@ZIF-8 nanoparticles was appropriate.

The antimicrobial activities of MH5C@ZIF-8 NPs primarily depended on the antibacterial peptides and zinc ions released to inflammation sites along with the hydrolysis and collapses of the whole frameworks (Ju et al., 2021; Wan et al., 2021). In detail, with the help of sustained released peptides and metal ions, the cell membranes, the membrane potentials, and the ion homeostasis were initially interrupted, causing obvious membrane dyshomeostasis, membrane permeability, and leakages of the cell component (Figure 1B) (Wen et al., 2021; Han et al., 2022). Afterwards, ions or peptides passed through the cell membranes to damage the intracellular nucleic acid, hamper the protein formations, block the ATP synthesis and inhibit the enzyme activity (Figure 1B) (Hou et al., 2021; Li et al., 2021). Additionally, the imidazole ring released from ZIF-8 might exhibit antibacterial effects as well by the disruption of the liposome composed of phospholipid containing the unsaturated fatty acid (Wan et al., 2021). As a consequence, *F. nucleatum*, *P. gingivalis*, and *S. mutans* would be almost all inactivated during these courses.

### 3.3 Cleaning capacity of mechanical-chemical synergistic treatments

**FIGURE 4:**
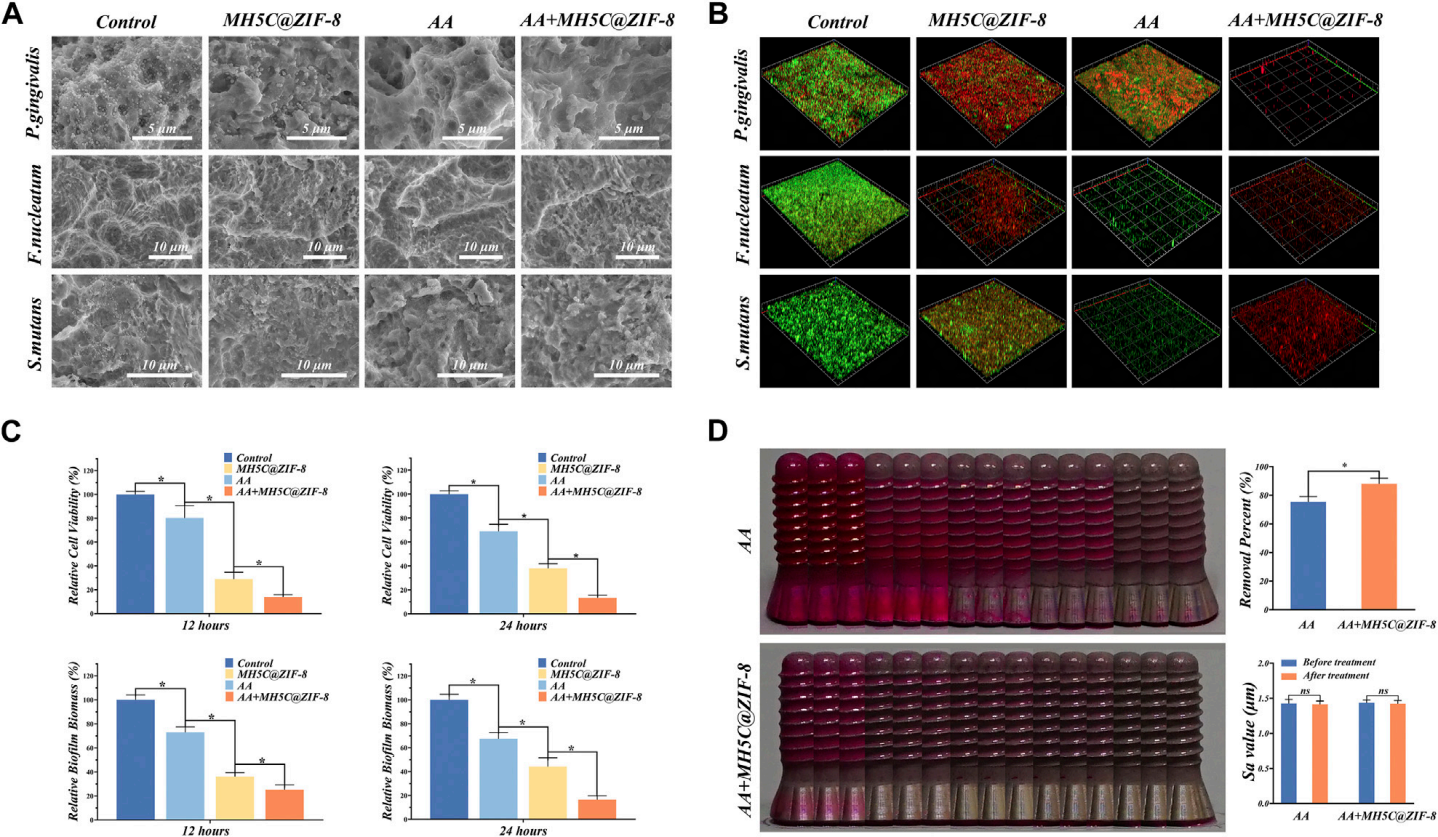
Decontamination efficiencies of the synergistic therapy using erythritol air-polishing coupled with MH5C@ZIF-8 NPs. **(A)** SEM morphologies of *F. nucleatum*, *P. gingivalis*, and *S. mutans* after different debridement. **(B)** Representative 3D live/dead images of single-species biofilms in different groups. **(C)** Bacterial activity and biofilm biomass in each group with different treatments. **(D)** Assessment of the residual stains from digital photo as well as the surface roughness after 60 s of air abrasion with or without the pre-treatment of MH5C@ZIF-8 NPs.

SEM observations were used to observe the morphologies and membrane integrities of three single-species biofilms. In the control group, the biofilms with the firm and dense cluster of internal bacteria were clearly visible (Figure 4A). Most bacteria in the control exhibited smooth and intact cell structures with abundant intracellular materials. In the group AA, structures of biofilms were disrupted into the disordered cluster, and the amounts of bacteria were evidently reduced, which might ultimately facilitate the transport and application of antimicrobial agents (Figure 4A). Accordingly, it was partly proved that the mechanical removals of biofilms remained the essential step to clean the infected surface during peri-implantitis treatments. In the MH5C@ZIF-8 group, most bacteria were distributed across the pores and merely the small piles instead of a whole piece of aggregated colony were observed (Figure 4A). Here, the cell wall of MH5C@ZIF-8-treated bacteria appeared dis-continuous and even collapsed. In the group AA + MH5C@ZIF-8, the pore on surfaces of titanium discs was exposed again and almost all the bacteria were collapsed, partially indicating that the strong clean capacities of the synergistic therapy (Figure 4A). Based on these, the air abrasion would disrupt the biofilm structures and remove the absorbed extracellular polymeric substances (EPSs) (Qin et al., 2020). During microbial biofilms formations, the secreted EPSs would ensure mechanical stabilities, form the three-dimensional scaffolds and facilitate bacteria adhesions (Wan et al., 2021; Yu et al., 2021). Hence MH5C@ZIF-8 NPs alone even at the high concentration of 125 μg/mL could not diffuse through the EPSs to kill the internal bacteria within polymicrobial biofilms. However, MH5C@ZIF-8 NPs could act as the adjunct to air abrasion successfully, which weakened the biofilms and killed the rest bacteria.

The typical three-dimensional images of live/dead staining for *F. nucleatum*, *P. gingivalis*, and *S. mutans* biofilms were depicted in Figure 4B, where red and green stained areas represented dead and live bacteria, respectively. In the control group, the majority emitted fluorescence while the minority emitted red fluorescence, suggesting that the excellent growth trends of internal bacteria (Figure 4B). In the group AA, a part of bacteria was dyed red, but lots of green fluorescence were still observed, indicating that antibacterial abilities of air abrasion on rough surfaces were relatively limited (Figure 4B). In the MH5C@ZIF-8 group, some dead bacteria were found, indicating that MH5C@ZIF-8 exhibited antibacterial properties against all three pathogenic bacteria (Figure 4B). In sharp contrast, in the group AA + MH5C@ZIF-8, only spotted and scattered red fluorescence has been observed, further verifying that the enhanced bactericidal activities of the synergistic therapy (Figure 4B). The antibacterial capabilities of most antimicrobial agents including MH5C@ZIF-8 alone were less effective against the mature biofilms (Kanwar et al., 2017; Renvert and Polyzois, 2018). For the above reason, the initial mechanical debridement for destroying the intact biofilms was quite essential (Qin et al., 2020). In this study, the pretreatment MH5C@ZIF-8 (125 μg/mL) followed by the air abrasion could fully break up the EPSs, efficiently penetrate the biofilms, and eventually eliminate the residual bacteria, thus the synergistic treatment might be an ideal way to manage biofilms around rough surfaces.

To evaluate the antimicrobial effects of the synergistic therapy on the mature polymicrobial biofilms formed on commercially used SLA titanium surfaces, we used the MTT assay and the CV staining assay to evaluate the relative biofilm biomass and the relative cell viability after different debridement. The relative cell viability after 12 h in groups MH5C@ZIF-8, AA and AA + MH5C@ZIF-8 was decreased to 80.31% ± 10.17%, 29.01% ± 5.67%, and 13.87% ± 1.98% (Figure 4C). The relative cell viability after 24 h in groups MH5C@ZIF-8, AA and AA + MH5C@ZIF-8 was decreased to 69.01% ± 5.74%, 38.07% ± 3.71%, and 13.24% ± 2.43% (Figure 4C). The relative biofilm biomass after 12 h in groups MH5C@ZIF-8, AA and AA + MH5C@ZIF-8 was decreased to 73.02% ± 4.54%, 36.02% ± 3.43%, and 25.12% ± 4.21% (Figure 4C). The relative biofilm biomass after 24 h in groups MH5C@ZIF-8, AA and AA + MH5C@ZIF-8 was decreased to 67.50% ± 5.01%, 44.21% ± 7.24%, and 16.59% ± 3.14% (Figure 4C). Air abrasion could destroy the structures of mature bacterial membranes, but it could not effectively kill internal bacteria in inaccessible areas (Louropoulou et al., 2014). Chemical decontaminations could kill individual bacteria, but it could not effectively penetrate the interior of mature polymicrobial biofilms (Ntrouka et al., 2011). Based our findings, MH5C@ZIF-8 at high concentrations (125 μg/mL) coupled with air abrasion effectively eliminated the polymicrobial biofilms remaining on previously contaminated implant surfaces.

### 3.4 Biofilm removal assessment using implant models

Mechanical debridement remained the gold standard in treatments of peri-implantitis (Sahrmann et al., 2015). Air abrasion employing erythritol powders exhibited excellent cleaning potentials on screw-shaped implants with rough surfaces in comparison with the manual or ultrasonic instrument (Al-Hashedi et al., 2017). Small particles like erythritol powders would reach the area inaccessible by large particles, and gain high solubility potentials that could reduce the amounts of the undissolved particle within the water-air mixtures, which finally might contribute to surface abrasions (Sahrmann et al., 2015; Tong et al., 2021). However, based on the digital photography, complete biofilm removals were not fulfilled on SLA surfaces even by air abrasion (Figure 4D). In other words, due to the complex and rough surfaces, mechanical decontaminations of these areas remained the challenging task in the managements of peri-implantitis. Quantitative data of the visual color analysis suggested significant biofilm removal differences between group AA + MH5C@ZIF-8 and group AA (Figure 4D). Here, with the pretreatment of MH5C@ZIF-8 (125 μg/mL), the biofilm removal percent was increased from 75.33% ± 3.78% to 88.01% ± 4.12% (Figure 4D). Previous studies have shown that the additions of antibacterial agents to small AA particles not only promoted the removals mature biofilms, but also decreased the likelihoods of the biofilm-associated infection in the future (Drago et al., 2017). Another study has demonstrated that chlorhexidine contained erythritol powders was quite effective to reduce bacterial viabilities on the infected titanium disc (Mensi et al., 2018). In this study, the surface roughness was not altered by the erythritol AA treatments with or without MH5C@ZIF-8 (Figure 4D). Hence it was safe to apply the synergistic therapy using erythritol AA coupled with MH5C@ZIF-8 NPs as it caused minimal changes in the surface roughness. Besides, glycine could be easily degradable by our body, so the presences of glycine particles seemed quite unproblematic. Therefore, the air abrasion associated with antimicrobial agents MH5C@ZIF-8 NPs favored the prognosis of peri-implantitis treatments.

### 3.5 Re-osseointegration potential after debridement

**FIGURE 5:**
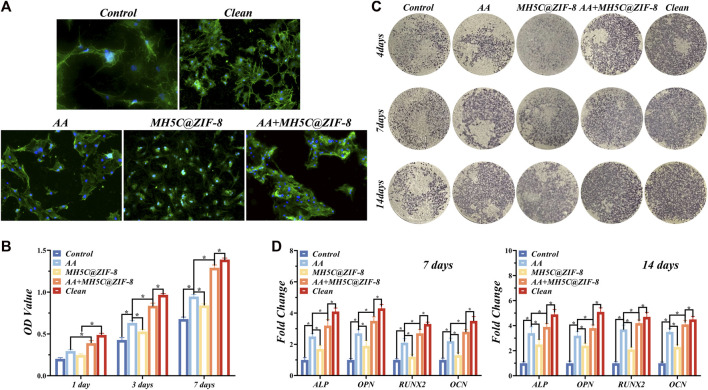
Re-osseointegration on contaminated titanium plates treated with erythritol air-polishing coupled with MH5C@ZIF-8 NPs. **(A)** Fluorescent images of BMSCs cultured on contaminated titanium plates after different treatments. **(B)** Cell proliferation assay of BMSCs cultured on contaminated titanium plates after different treatments. **(C)** ALP staining of BMSCs cultured on contaminated titanium plates after different treatments. **(D)** Osteogenic-related gene expression after culturing for 7 and 14 days.

The initial cell adhesions were observed by staining by DAPI and FITC for the visualization of the nuclei and F-actin. The spherical morphology of BMSCs was observed on groups control, AA and MH5C@ZIF-8 owing to a lack of the filopodia extension (Figure 5A). The expression of F-actin in the group AA + MH5C@ZIF-8 was more obvious and lots of filopodia were detected accordingly (Figure 5A). The BMSCs on these totally clean surfaces demonstrated the well-organized cytoskeleton structure, the multipolar spindle morphologies, and a lot of lamellipodia as well as filopodia (Figure 5A). The results of CCK-8 assay were remarkably consistent with the adhesive results. The BMSC viabilities in groups clean and AA + MH5C@ZIF-8 were significantly higher than in other groups at all timepoints (Figure 5B). The average OD value increased along with the culture time suggesting the continuous cell proliferations in the group AA + MH5C@ZIF-8 (Figure 5B). The cell adhesion and cell proliferation experiments consistently indicated that the AA + MH5C@ZIF-8 would debride most of the biofilms and the potential residual particles showed no apparent cytotoxicity to the proliferation of BMSCs. Here, to evaluate the effects of contaminated surfaces after debridement on cell behaviors, we maintained surface morphologies of contaminated surfaces by sterilization before cellular studies, which have been performed by other researchers before (John et al., 2014; Qin et al., 2020). The ultimate fates of titanium implants depended on outcomes of the so-called “race for the surface” between the oral pathogen and the host cell (Zhu et al., 2017). Implant surface contaminations mainly caused two corruptive effects, one was the facilitated recolonizations of pathogenic bacteria, and the other was the reduced cytocompatibilities of eucaryotic cells involved in healing processes (Sahrmann et al., 2015). Here, after incubations with these three bacteria species for 2 days, the original microscopic pore on SLA surfaces was totally covered with numerous bacteria as well as EPSs, thus establishing mature multispecies biofilms. After debridement, pores on contaminated surfaces were distinguished again, which facilitated the adhesion of osteogenic cells. Our results showed that BMSCs acclimated and grew rapidly in the AA + MH5C@ZIF-8 group, which indicated that the retained biofilms after the synergistic therapy did no harm to the initial adhesions and proliferations of BMSCs. This might be due to high clean capacities of the synergistic therapy and the residual particles would not inhibit normal cell functions.

The ALP staining images of BMSCs in different groups were displayed in Figure 5C. The previously contaminated discs in groups AA or MH5C@ ZIF-8 exhibited increased ALP expressions as compared to the control group (Figure 5C). Among all groups, expression of ALP was highest in the clean group, followed by the Group AA + MH5C@ZIF-8 (Figure 5C). Furthermore, RT-PCR results showed that after culturing for 7 and 14 days, obvious increased mRNA levels of ALP, OPN, RUNX2, and OCN were observed in the groups AA or MH5C@ ZIF-8 as compared with the control (Figure 5D). It was also noted that BMSC osteogenesis in group AA + MH5C@ZIF-8 was similar to those on intact clean titanium surface (Figure 5D). These results might be ascribed to the destructions and removals of the biofilms and the subsequent re-exposures of the micropores on SLA surfaces in the group AA + MH5C@ZIF-8, which improved the osteogenic differentiation of BMSCs. These findings also suggested that the residual biofilm after the sterilization in the group AA + MH5C@ZIF-8 caused no adverse effects on the cell behaviors. Moreover, the enhanced BMSCs osteogenic differentiations observed in the group AA + MH5C@ZIF-8 might be owing to the formations and precipitations of zinc ions from ZIF-8 nanoparticles (Hoseinpour and Shariatinia, 2021). Here, ZIF-8 NPs in the culture medium agglomerated and precipitated frequently on the SLA surfaces after the pretreatment of MH5C@ZIF-8 for 24 h, which was somewhat similar to ZIF-8 coatings on titanium surfaces (Liu et al., 2020b). Previous studies exhibited that the functionalized ZIF-8 coatings deposited on titanium promoted the viabilities and the osteogenic property of osteogenic cells (Sandomierski et al., 2022). Another study also demonstrated that ZIF-8-coated titanium surfaces enhanced the osteogenesis of the osteoblast and promoted new bone formations (Zhang et al., 2017). Nevertheless, too high concentrations of ZIF-8 still might bring up somewhat toxic effects to cells. Therefore, our findings emphasized the great potential of the synergistic therapy as a credible alternative for improving the biocompatibility and rendering the re-osseointegration on contaminated implant surfaces, boding well for the comprehensive applications in peri-implantitis treatments.

However, our study is preliminary. First, the specific antibacterial mechanisms of MH5C@ZIF-8 NPs retained indistinct and further molecular mechanism experiments are still needed. Second, during cellular experiments, only dead biofilms were maintained on titanium surfaces and these circumstances did not reflect the condition occurring clinically. Further animal experiments should be designed to evaluate re-osteogenesis effects of the synergistic therapy.

## 4 Conclusion

To summarize, an effective and mild method to synthesis the antibacterial agent MH5C@ZIF-8 NPs has been established. The formed MH5C@ZIF-8 NPs possessed nanoscale sizes as well as robust stabilities, and effectively protected MH5C from rapid release. The MH5C@ZIF-8 NPs not only possessed slow-release abilities, but also exhibited excellent antibacterial abilities against pathogenic bacteria. Here, the air abrasion with erythritol powders was safe to use on implant surfaces as it resulted in obvious biofilm destructions and minimal surface roughness changes. Moreover, their cleaning capacities was significantly increased with the pretreatment of MH5C@ZIF-8 NPs. However, none of MH5C@ZIF-8 NPs nor erythritol air abrasion alone was able to completely remove the stained biofilms on titanium. In contrast, the synergistic strategy using erythritol air abrasion coupled with an as-synthesized pH-responsive antimicrobial agent MH5C@ZIF-8 NPs demonstrated excellent debridement and re-osteogenesis properties. In other words, notwithstanding the limitations of our study, MH5C@ZIF-8 NPs at high concentrations combined with air abrasion significantly removed polymicrobial biofilms that remained on previously contaminated titanium surfaces. The osteogenic potentials of BMSC were also regained on titanium surfaces treated by the synergistic therapy *in vitro*, which might provide a new idea for the treatments of peri-implantitis.

## Data Availability

The original contributions presented in the study are included in the article/Supplementary Material, further inquiries can be directed to the corresponding authors.
